# A Latent Activated Olfactory Stem Cell State Revealed by Single Cell Transcriptomic and Epigenomic Profiling

**DOI:** 10.1101/2023.10.26.564041

**Published:** 2023-10-31

**Authors:** Koen Van den Berge, Hsin-Jung Chou, Divya Kunda, Davide Risso, Kelly Street, Elizabeth Purdom, Sandrine Dudoit, John Ngai, Whitney Heavner

**Affiliations:** 1 Statistics and Decision Sciences, Janssen R&D, Beerse, Belgium; 2 Department of Molecular and Cell Biology, University of California, Berkeley, CA; 3 Molecular Neurobiology Section, National Institute of Neurological Disorders and Stroke, National Institutes of Health, Bethesda, MD; 4 Department of Statistical Sciences, University of Padova, Padova, Italy; 5 Division of Biostatistics, Department of Population and Public Health Sciences, Keck School of Medicine, University of Southern California, Los Angeles, CA; 6 Department of Statistics, University of California, Berkeley, CA; 7 Division of Biostatistics, School of Public Health, University of California, Berkeley, CA

## Abstract

The olfactory epithelium is one of the few regions of the nervous system that sustains neurogenesis throughout life. Its experimental accessibility makes it especially tractable for studying molecular mechanisms that drive neural regeneration after injury-induced cell death. In this study, we used single cell sequencing to identify major regulatory players in determining olfactory epithelial stem cell fate after acute injury. We combined gene expression and accessible chromatin profiles of individual lineage traced olfactory stem cells to predict transcription factor activity specific to different lineages and stages of recovery. We further identified a discrete stem cell state that appears poised for activation, characterized by accessible chromatin around wound response and lineage specific genes prior to their later expression in response to injury. Together these results provide evidence that a subset of quiescent olfactory epithelial stem cells are epigenetically primed to support injury-induced regeneration.

## Introduction

The generation of cellular diversity in the nervous system requires specification of discrete cell lineages from multipotent neural progenitor cells. Neural tissues that are uniquely capable of regenerating multiple lineages throughout adulthood often undergo frequent cellular turnover under both homeostatic conditions and in response to injury. Regenerative capacity in the nervous system therefore requires (1) neural progenitor cell maintenance, (2) the ability to specify multiple discrete lineages, and (3) the ability to respond to acute injury.

The olfactory epithelium (OE) is one of the few sites in the nervous system that supports active neurogenesis throughout life (reviewed in (Denans, Baek, and Piotrowski 2019)). The OE divides these capacities between two different progenitor cell populations. Under normal homeostatic conditions, the differentiation of globose basal cells (GBCs), which are the actively proliferating neural progenitor cells in the OE stem cell niche, sustains lifelong olfactory neurogenesis (Caggiano, Kauer, and Hunter 1994) (J. E. Schwob et al. 1994) (Graziadei and Graziadei 1979). Upon injury, however, horizontal basal cells (HBCs), the normally quiescent stem cells of the OE, self-renew and differentiate to replace lost neurons, neural progenitors, and other cell types including the sustentacular or support cells, a process requiring the production of multiple cell types in concert in order to repair the damaged system (James E. Schwob, Youngentob, and Mezza 1995) (Leung, Coulombe, and Reed 2007) (Iwai et al. 2008). The tumor suppressor *Trp63*, which is expressed exclusively in HBCs, is downregulated in response to injury. This downregulation is required for HBCs to differentiate into other cell types, suggesting that *Trp63* acts as a gatekeeper to promote HBC quiescence while inhibiting HBC differentiation under homeostatic conditions (Fletcher et al. 2017, 2011; Schnittke et al. 2015; A. Packard et al. 2011).

We previously investigated mechanisms involved in lineage specification during OE homeostasis (Fletcher et al. 2017) and renewal after injury (Gadye et al. 2017) using single-cell RNA sequencing with in vivo lineage tracing of HBCs and their descendants. These studies identified the early HBC transition states during which distinct cell fates are specified and pointed to several signaling pathways that regulate whether a cell will self-renew or follow the neuronal or sustentacular lineage. Lineage tracing of *Hopx-*expressing HBCs after injury produced only sustentacular and neuronal cells but no HBCs (Gadye et al. 2017), suggesting that olfactory epithelial cell fate decisions are made in the activated HBC state.

The pathways that regulate the rapid transition of HBCs from a resting to an activated state have yet to be characterized. There is some evidence to suggest a role for epidermal growth factor receptor (Egfr) signaling in HBC activation (Z.-H. Chen et al. 2020; Gadye et al. 2017) and a role of canonical Wnt signaling in the transition of HBCs from a resting to an activated state as well as promoting the neuronal lineage over sustentacular and other cell fates (Y.-Z. Wang et al. 2011) (M. Chen et al. 2014) (Fletcher et al. 2017). Interestingly, the proliferative stem cell transcription factor SOX2 was found to be required for neuronal cell fate competence in the OE but was not required for HBC self-renewal or sustentacular cell fate (Y.-Z. Wang et al. 2011; Gadye et al. 2017). In the absence of SOX2, cells in the neuronal lineage arising from activated HBCs appeared to arrest prior to the formation of GBCs (Gadye et al. 2017), while over-expression of SOX2 expanded the GBC pool (A. I. Packard, Lin, and Schwob 2016). Together, these studies begin to illustrate how various signaling pathways and transcription factors individually contribute to HBC activation and cell fate decisions, but how these collectively operate in regulatory networks to coordinate specification of different lineages is unknown.

Advances in single-cell profiling have shed light on the role of chromatin accessibility in cell plasticity, cell fate potential, and functional heterogeneity within progenitor cell populations and have improved the ability to predict gene regulatory networks (Trevino et al. 2021) (Ma et al. 2020) (reviewed in (Shema, Bernstein, and Buenrostro 2019)). Here, we have leveraged droplet-based single-cell sequencing to assess the epigenomic and transcriptomic states of individual lineage-traced cells arising from HBCs during recovery from acute injury. With these datasets we identified the downstream transcription factor cascades associated with OE regeneration. Intriguingly, we detected a subpopulation of quiescent and activated HBCs that exhibit genes residing in accessible chromatin prior to their later expression in response to injury, suggesting the existence of a latent activated stem cell state poised to support rapid regeneration following injury to the epithelium. Collectively, our findings contribute to an understanding of the general principles governing neural stem cell maintenance and injury-induced repair of the nervous system.

## Results

### Reconstructed lineages from single cells reveal dynamic transcriptional response to injury

**Figure 1:**
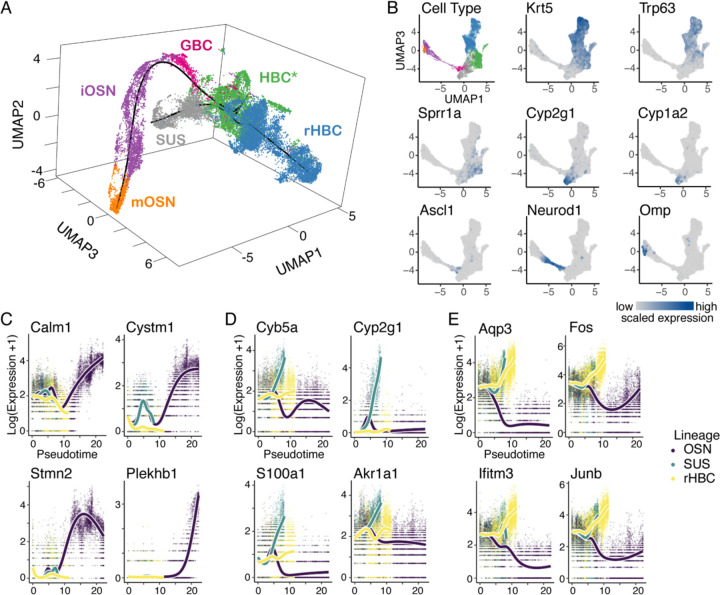
Trajectory inference and differential expression analysis of lineage-traced scRNA-seq data. **(A)** Inferred trajectory in 3D UMAP space, with cells colored according to cell type. Starting from the HBC* population, the trajectory consists of three lineages developing into rHBC, Sus, and mOSN cells. **(B)** Cells in 2D UMAP space, colored according to cell type (top left panel) or the expression of known markers (all other panels), grey denoting no/low expression and blue denoting high expression. The top cells in the UMAP represent the rHBC lineage, the lefthand cells the neuronal lineage, and the bottom cells the sustentacular lineage. **(C-E)** Markers for each lineage identified by differential expression for the neuronal (C), sustentacular (D), and rHBC lineage (E).

To investigate the transcriptional response to injury at the cellular level in thousands of cells, the olfactory epithelium of *Krt5-CreER(T2); Rosa26*^*eYFP/eYFP*^ adult (age 3–7 weeks) mice (23 total; 15 F and 8 M) was injured using methimazole administration, and cells were sampled at 24h, 48h, 96h, 7 days, and 14 days after injury, as previously described (Gadye et al. 2017; Brann et al. 2020). A total of 25,469 cells were assayed using the 10x Genomics Chromium v2 protocol for single-cell RNA sequencing (scRNA-seq). After removal of doublets, microvillous cells, respiratory epithelial cells, and other manually selected cells that were asynchronous with respect to chronological time or separate from the main differentiation process of interest (i.e., mature neurons present at 24h and 48h post-injury), 20,426 cells remained (dataset as described in Brann et al. 2020). Uniform Manifold Approximation and Projection (UMAP) dimensionality reduction (McInnes, Healy, and Melville 2018) revealed structure that was clearly correlated to the chronological time of the sampled cells (Supplemental Figure 1), indicating that the injury triggered a dynamic transcriptional response underlying differentiation. Cell types identified by manual annotation of clusters using expression of known marker genes included activated horizontal basal cells (HBCs*), regenerated HBCs (rHBCs), sustentacular cells (Sus), globose basal cells (GBCs), immature olfactory sensory neurons (iOSNs), and mature olfactory sensory neurons (mOSNs) (Figure 1a,b).

In order to infer the developmental trajectory of HBC progeny, we used *slingshot* (Street et al. 2018) on UMAP reduced-dimensional space, which revealed a trajectory consisting of three main lineages. Starting from HBCs*, these lineages produced rHBCs, Sus cells, and mOSNs (via GBCs and iOSNs) (Figure 1a, black line). To distinguish these lineages at the transcriptomic level, gene expression along the trajectory was explored. Trajectory-based differential expression analysis using *tradeSeq* (Van den Berge et al. 2020) revealed genes significantly upregulated in each of the lineages (Figure 1c-e, Supplemental Figure 2). The neuronal lineage genes Calmodulin (*Calm1*), Stathmin2 (*Stmn2*), and Plekstrin Homology Domain Containing B1 (*Plekhb1*) were functionally consistent with sensory neurons. Moreover, while we previously found that Sus and rHBC lineages have similar gene expression profiles (Fletcher et al. 2017), in this larger dataset we found that the Sus lineage had particularly high expression of genes involved in oxidation-reduction, whereas the rHBC lineage was enriched for the activity responsive genes *Fos* and *Junb*. Interestingly, a particularly large set of genes (5,325) was uniquely involved in the neuronal lineage (Supplemental Figure 3), possibly reflecting the complex transcriptional programs required for neurogenesis. Around 1,200 genes were found to be common to all lineages.

### Leveraging smooth gene expression profiles to uncover transcription factor activity cascades

The large number of genes involved in regeneration of the OE upon injury suggests that coordinated gene regulatory mechanisms specify distinct cell fates after HBC activation. To further advance our comprehension of this complex process, we aimed to understand the regulatory networks involved in each lineage, focusing on a set of 1,532 mouse transcription factors (TFs) selected previously (Fletcher et al. 2017). Leveraging the fitted gene expression functions along pseudotime from the negative binomial generalized additive models (NB-GAM) implemented in *tradeSeq* (Van den Berge *et al.* 2020), we developed a workflow to discover TFs that are involved in the differentiation of each lineage (see Methods). The goal of our approach was to infer which TFs are significantly increasing in expression along each developmental lineage. To do so, we tested whether the first derivative of their fitted expression function was significantly greater than an arbitrary threshold. This allowed us to 1) assess which TFs were significantly peaking at some point along a lineage and 2) derive at which point along differentiation each TF was most active, which here we assume to correspond to the most statistically significant increase in expression. We have made this functionality available in *tradeSeq*.

**Figure 2:**
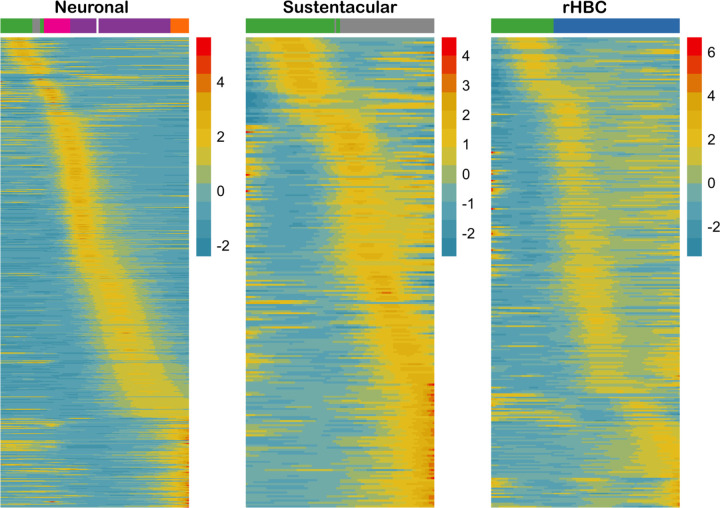
Inferred transcription factor activity cascade for each of the three lineages in the trajectory. Heatmaps of fitted expression measures from tradeSeq, where the x-axis for each panel represents 100 equally-wide pseudotime bins for a given lineage, and the most abundant cell type in each bin is indicated using the colorbar at the top of the heatmap (colors correspond to those in Figure 1a; If there are too few cells in a bin, no color is provided). Each row in each heatmap represents the expression of a transcription factor, normalized to zero mean and unit variance within a lineage. The TFs are ordered according to the pseudotime of their most significant peak, uncovering a TF activity cascade within each lineage.

The present analysis uncovered TF activity cascades with distinct sequential activational patterns for each of the major olfactory epithelium cell lineages (Figure 2). The number of TFs contributing to the cascade was 524, 231, and 284 for the neuronal, sustentacular, and rHBC lineages, respectively. The large discrepancy in the number of TFs considered most active between the neuronal lineage and the other two lineages likely reflects the complexity of the transcriptional programs involved in the neuronal lineage and is consistent with our previous observation that Sus and HBC lineages have similar gene expression profiles.

By searching for TFs with an expression peak at a similar pseudotime in different lineages (see Methods), we found that 19 TFs were common to all three lineages at the same point in pseudotime (Supplemental Figure 4a; Supplemental File 1).

We next hypothesized that the TFs categorized as most active in a particular lineage would reveal lineage-specific developmental processes. To identify such lineage-enriched TFs, we prioritized (see Methods) a list of 352, 31, and 61 TFs that we found to be most active only in the mOSN (e.g., *Neurog1*), Sus (e.g., *Sec14l2*), and rHBC (e.g., *Trp63*) lineages, respectively (Supplemental Figure 4b-d; Supplemental File 1). The TF activity cascade allowed us to group TFs involved in each lineage according to the moment within the lineage in which they were most active and thus infer which cellular processes were activated over time. Gene set enrichment analysis of these groups illuminated the processes these TFs activated at various stages of differentiation. For example, along the neuronal lineage, an initial stress response was observed at the early HBC* stage, which was followed by cell cycle regulation and neuron differentiation during the GBC and iOSN stages. A large group of TFs at the iOSN and mOSN stages were involved in processes such as dendrite development, cell projection, and calcium mediated signaling (Supplemental Figure 4e).

### Prediction of TF activity through deconvolution of mRNA expression

**Figure 3:**
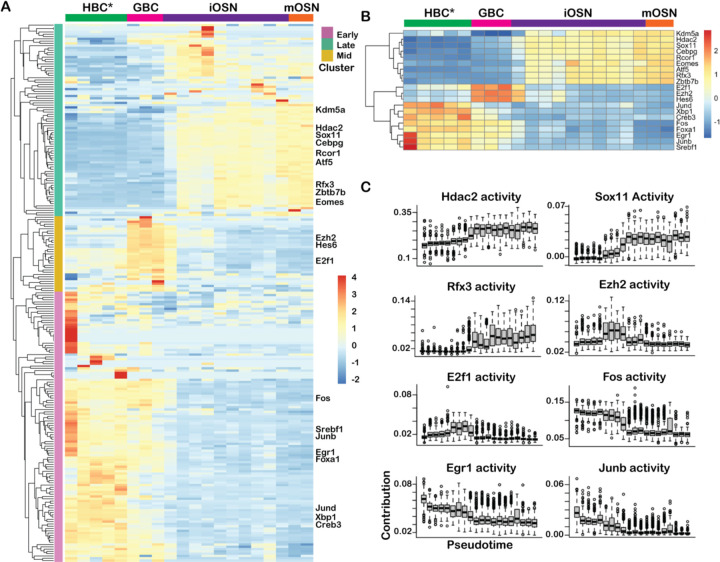
Deconvolution of gene expression reveals dynamic TF activity along the neuronal lineage. **(A)** Clustered heatmap of scaled activity for TFs (rows) that are differentially active along the neuronal lineage. The x-axis represents pseudotime and the dominant cell type in each pseudotime bin is indicated at the top of the heatmap. The TFs are clustered into three groups (early, mid, and late activity) using hierarchical clustering. **(B)** Clustered heatmap of scaled TF activity for top 20 most variable TFs. **(C)** Change in TF activity over pseudotime for several individual TFs from each cluster.

To further evaluate the activity of TFs during neuronal regeneration, we resolved the collective change in gene expression over pseudotime along the neuronal lineage (i.e., HBC*, GBC, iOSN, and mOSN stages) into co-regulated gene groups (Figure 3a). First, for each cell in the lineage and each of the 352 mOSN lineage TFs, we probabilistically assigned mRNAs to the TFs that most likely drove their expression. We then predicted TF activity based on the number of mRNAs likely driven by each TF within bins of pseudotime (see Methods). This revealed 3 clusters of TFs that were most active either at the HBC* (early), GBC (mid), or OSN (late) stage. A closer look at the 20 most variable TFs (Figure 3b) revealed that the immediate early genes *Egr1*, *Fos*, *Junb*, and *Jund* and the pioneer TF *Foxa1* were most active early in the lineage, while *Hes6*, *Ezh2*, and *E2f1* were most active at the GBC stage, and several differentiation genes, including *Sox11* and *Rfx3*, were most active late in the lineage, at the OSN stage (Figure 3c).

### Chromatin accessibility upon injury reveals transcriptional priming

Our prediction of robust transcription factor cascades associated with each lineage suggests that dynamic gene regulatory networks are initiated in activated HBCs. However, our earlier finding that TF expression was similar between resting and activated HBCs despite significant differential expression of ~2,000 genes (Gadye *et al.* (2017)) suggests that HBCs are epigenetically primed to initiate broad rapid changes in gene expression without immediate large changes in TF expression, similar to other stem cell types, such as muscle stem cells (Rodgers et al. 2014), resting CD4+ T cells (Z. Wang et al. 2009; Rogers et al. 2021), and hepatocytes (Reizel et al. 2021). To test this hypothesis and to begin to identify gene regulatory networks that are initiated upon injury, we performed an assay for transposase accessible chromatin with sequencing (ATAC-seq) (Buenrostro et al. 2013; Corces et al. 2017) on ICAM1+ HBCs isolated either from uninjured olfactory epithelium or 24 hours after injury with methimazole. We also generated bulk RNA sequencing libraries to serve as a reference for gene expression.

**Figure 4:**
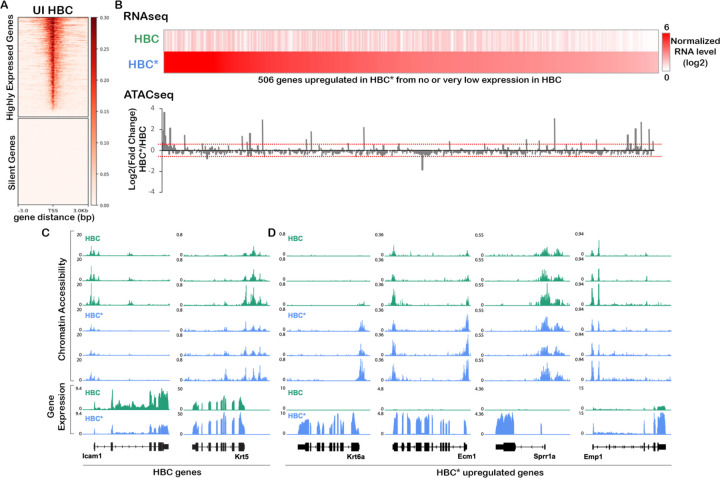
Early response genes are primed for activation at the chromatin level. **(A)** Heatmaps of ATAC-seq read counts from uninjured (UI) HBCs around the TSS of highly expressed genes (top) or silent olfactory receptor genes (bottom). **(B)** Heatmap of normalized bulk gene expression values (RPKM) in resting HBCs versus activated HBCs (HBC*) for the top 506 genes that are upregulated in HBCs after injury, with genes ordered from left to right according to descending expression in activated HBCs (top). Bar graph showing the log2(fold-change) in chromatin accessibility after injury relative to before injury (bottom), where dotted lines indicate log2(fold-change) of 0.5 and −0.5. **(C,D)** ATAC-seq (top) and bulk RNA-seq (bottom) read counts before (green) and after (blue) injury around genes that are known to decrease (Icam1) or increase (Krt5) in expression in injured HBCs (C) and wound response genes (Krt6a, Ecm1, Sprr1a, and Emp1) (D).

To identify epigenetic changes associated with injury, we first asked whether gene expression was associated with chromatin accessibility in resting HBCs. We established two gene sets for resting HBCs: 1) genes that are highly expressed in HBCs and 2) olfactory receptor genes, which are not expressed (“silent”) in HBCs. We then assessed chromatin accessibility in the regions surrounding these genes’ transcriptional start sites (TSS) in resting HBCs (Figure 4a). As expected, regions surrounding the TSS of highly expressed genes were found in accessible chromatin, whereas those of silent genes did not appear in accessible chromatin, demonstrating that the TSS of silent genes generally occupies inaccessible chromatin.

Next, the bulk RNA-seq data were used to identify 506 “early response” genes that are upregulated in activated HBCs and had no or very low expression in resting HBCs. We then quantified changes in chromatin accessibility around each gene’s TSS between resting and activated states. We found that these early response genes tended to have comparable accessibility around the TSS before and after injury despite significant increases in gene expression, suggesting that some of these genes may be primed for rapid activation upon injury (Figure 4b). We next looked at chromatin changes after injury over the gene bodies of individual marker genes for HBCs (*Icam1* and *Krt5*) and wound response genes (*Krt6a*, *Ecm1*, *Sprr1a*, and *Emp1*). We found that *Icam1* lost and *Krt5* gained chromatin accessibility consistent with changes in gene expression upon injury. Similarly, the chromatin around the wound response gene *Krt6a* became accessible exclusively when activated. However, other wound response genes already possessed partially (e.g., *Ecm1*) or fully (e.g., *Sprr1a* and *Emp1*) accessible chromatin prior to HBC activation, suggesting that they may be primed for activation upon injury (Figure 4c,d).

### Single cell analysis of chromatin accessibility reveals multiple HBC states

**Figure 5:**
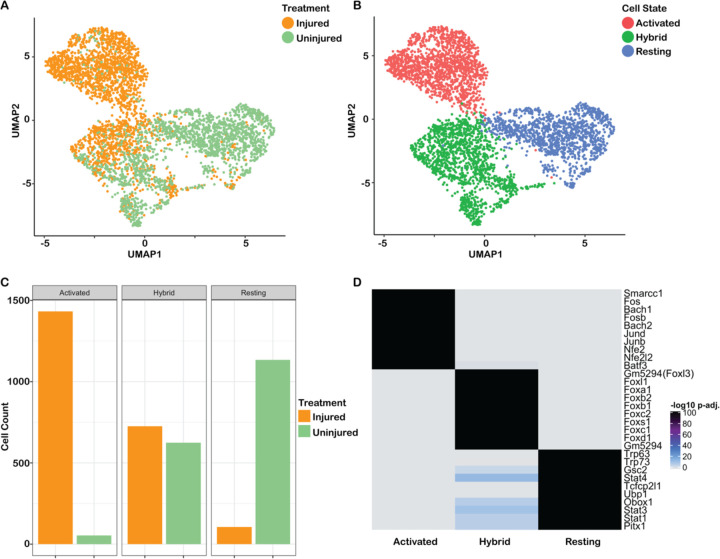
scATAC-seq data uncover three HBC states. **(A-B)** UMAP dimensionality reduction of the scATAC-seq data; cells are colored according to treatment (A) or according to state (B). **(C)** Barplot visualizing the number of (un)injured cells in each cluster. **(D)** Heatmap (unclustered) showing the Benjamini-Hochberg FDR adjusted p-values for the top 10 enriched TF motifs (rows) for each cell state (columns). The heatmap clearly illustrates that the three sets of TFs are high/insignificant (grey) in specific HBC groups and low/significant (black) in others.

To assess whether injury response genes are epigenetically primed in all or a subset of resting HBCs, we performed single-cell ATAC-seq on lineage-traced FACS purified HBCs (*Krt5-CreER(T2)*; *Rosa26*^*eYFP/eYFP*^) before and 24 hours after injury with methimazole. Chromatin accessibility profiles of 5,743 cells were obtained. Gene activity scores were estimated using the ArchR framework for scATAC-seq (Granja et al. 2021). After quality control and doublet removal, 4,732 cells remained. Batch integration was performed using Harmony (Korsunsky et al. 2019), and the batch-corrected Harmony embeddings were used as input to UMAP dimensionality reduction for visualization. Clustering on the Harmony embeddings identified the major cell types that were present in the scRNA-seq data. HBCs made up the largest cluster, and neuronal and sustentacular cells each made up a relatively small clusters (Supplemental Figure 5), as identified by the open chromatin state of their respective marker genes (Supplemental Figure 6). Interestingly, the subset of 4,076 HBCs itself seemed to consist of three distinct clusters, possibly corresponding to different HBC states (Figure 5a). Two clusters consisted almost exclusively of either injured or uninjured cells, which we hypothesize corresponded to activated and resting states, respectively, as previously reported (Gadye et al. 2017; Fletcher et al. 2017) (Figure 1). However, a third cluster contained nearly equal numbers of injured and uninjured cells (Figure 5b,c), which may reflect a distinct epigenetic state shared by both resting and activated cells. Given the mixture of injured and uninjured cells, we labeled this third cluster “hybrid.”

To determine whether the three clusters indeed represent distinct HBC states, we looked for regions of accessible chromatin that distinguished each cluster. Peak calling using MACS2 (Zhang et al. 2008) on the three HBC clusters resulted in a set of 183,712 peaks. We identified marker peaks for each of these clusters using ArchR (see Methods), uncovering 12,146, 8,492, and 3,513 marker peaks for the injured, hybrid, and uninjured clusters, respectively (nominal FDR <= 0.01 and log_2_FC > 1.25). The cisbp database (Weirauch et al. 2014) was used to derive transcription factor motifs, which were subsequently assessed for enrichment in these sets of marker peaks (Figure 5d). The results reflected TFs hypothesized to play a role in HBC activation or quiescence: motifs for the activity-responsive TFs Fosb and Junb were enriched in the activated state, while motifs for the canonical resting HBC TFs Trp63 and Trp73 were enriched in the resting state. It is interesting to note that motifs for Smarcc1, the main regulatory component of the Brg1-associated factor (BAF) chromatin remodeling complex important for early stem cell differentiation, cell fate determination, and keratin gene expression (Lim et al. 2020; Schaniel et al. 2009), were enriched in activated HBCs. Remarkably, the top upregulated motifs for the hybrid cell state were all members of the Forkhead box (Fox) family of TFs, possibly reflecting an HBC gene regulatory network consisting of Fox TFs.

### Early response genes are epigenetically primed in a subset of resting HBCs

**Figure 6:**
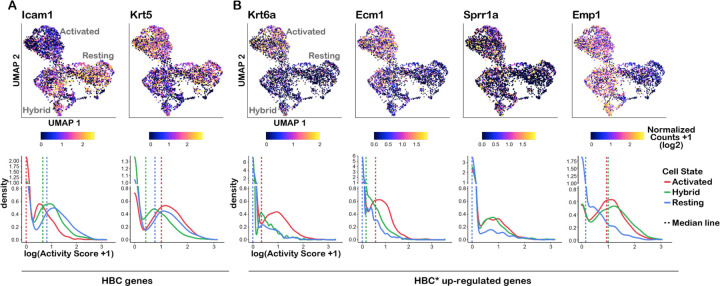
A subset of injury response genes are primed for activation in a subset of resting HBCs. **(A)** Two-dimensional UMAP representation of scATAC-seq data where cells are colored according to gene activity scores for HBC marker genes lcam1 and Krt5 (top) and density plots of gene activity scores for Icam1 and Krt5 in 3 scATAC-seq clusters representing 3 HBC states (bottom). **(B)** UMAP and density plots of gene activity scores for wound response genes.

The scATAC-seq results suggest that there are at least two states comprising HBCs from uninjured olfactory epithelium (“resting” and “hybrid”), while the bulk ATAC-seq results (Figure 4) suggest that the chromatin around some silent injury response genes is accessible in resting HBCs. To assess in which state(s) these genes are accessible, we compared the accessibility of four early response genes between the three scATAC-seq clusters using the gene activity scores from ArchR. We visualized the normalized accessibility for each gene across individual cells in reduced dimensional space and plotted the distribution of activity scores for each cluster by density plot (Figure 6, Supplemental Figure 7).

As a positive control, we first assessed chromatin accessibility for the HBC marker genes *Icam1* and *Krt5* (Figure 6a). *Icam1* was most accessible in the resting HBC cluster, and appeared to be least accessible in injured HBCs, consistent with its downregulation upon injury. Similarly, *Krt5* was most accessible in activated HBCs, as was *Krt6a*, again consistent with the bulk ATAC-seq results and their upregulation in expression upon injury (Figure 6a,b and Supplemental Figure 7a). Among the wound response genes, *Ecm1* was most accessible in the activated cluster, but more accessible in the hybrid cluster compared with the resting cluster. *Sprr1a* and *Emp1*, which the bulk ATAC-seq data predicted to be highly accessible in resting HBCs, were approximately equally accessible in the activated and hybrid clusters (Figure 6b). The finding that all four wound response genes were least accessible in the resting cluster suggests that only a subset of resting HBCs (the “hybrid” HBCs) are primed to activate a subset of wound response genes that occupy highly accessible chromatin prior to injury.

### Integration of scRNA-seq and scATAC-seq data suggests a latent activated olfactory stem cell state

**Figure 7:**
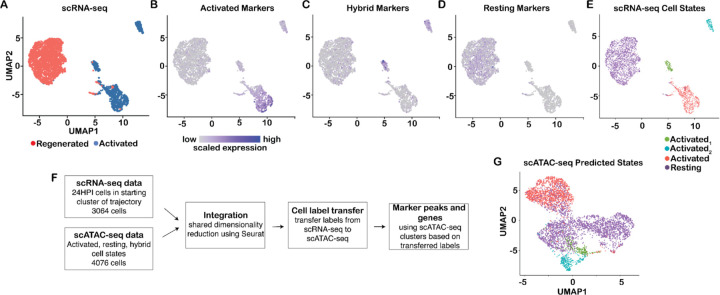
Integration of scRNA-seq and scATAC-seq data. **(A-D)** scRNA-seq data of activated and regenerated HBCs visualized in UMAP space. (A) scRNA-seq data with cells colored according to cell type. (B-D) scRNA-seq data with cells colored according to expression of genes that were found to be markers for each of the three cell states identified using scATAC-seq gene activity scores. ‘Activated’ and ‘Resting’ genes identified using scATAC-seq are correspondingly upregulated in scRNA-seq data for activated cells (B) and regenerated cells (D), while ‘Hybrid’ genes are upregulated in the two small activated subclusters (C). The expression values were first scaled within each gene to have zero mean and unit variance across all cells, upon which the scaled expression was summed across genes within each cell. **(E)** scRNA-seq data with cells colored according to cell state. **(F)** Workflow for cell label transfer from scRNA-seq to scATAC-seq data. First, Seurat was applied to integrate the scRNA-seq and scATAC-seq data by shared dimensionality reduction using canonical correlation and to transfer the scRNA-seq cell labels to the scATAC-seq dataset. Next, the transferred labels were used to define marker peaks and genes in the scATAC-seq dataset. **(G)** scATAC-seq data with cells colored according to cell state predicted by transferring cell labels from scRNA-seq data.

To better characterize the three different HBC epigenetic states, we further explored the agreement between the scRNA-seq and scATAC-seq data (Figure 7) using shared dimensionality reduction and cell label transfer as implemented in the Seurat software (Stuart et al. 2019) (see Methods). First, for the scRNA-seq data, we focused on cells that were sampled at 24 hours post injury (HPI) and clustered at the starting point of the trajectory (i.e., contained within the cluster that was assigned as the starting cluster) and regenerated HBCs. Interestingly, a reduced-dimensional representation of these gene expression data revealed three clusters of activated HBCs, while regenerated HBCs formed a single cluster, consistent with results from the scATAC-seq data showing multiple activated HBC states (Figure 7a). However, while the scRNA-seq data showed multiple states only for activated HBCs, the scATAC-seq data showed multiple states for both resting and activated HBCs.

Next, we compared the transcriptomic and epigenetic datasets by identifying gene activity markers for each of the scATAC-seq clusters and mapping them onto the scRNA-seq HBC clusters. Importantly, we first confirmed that both injured and uninjured cells contributed to the gene activity markers of the hybrid cell state (Supplemental Figure 8a). We then visualized the ATAC-seq-identified marker genes of “activated” and “resting” HBCs in the scRNA-seq data, which revealed upregulation of marker gene expression in the analogous (“activated” and “resting”) scRNA-seq cell populations (Figure 7b,d), linking these cell states in epigenetic and transcriptomic space. Surprisingly, the “hybrid” marker genes were enriched in the two smaller scRNA-seq clusters of activated HBCs, suggesting that “hybrid” genes, while accessible in both injured and uninjured cells, are only expressed upon injury in a subset of activated HBCs (Figure 7c).

Given this link between accessible chromatin and gene expression, we used the Seurat workflow (Stuart et al. 2019) to integrate the scRNA-seq and scATAC-seq datasets as follows: after shared dimensionality reduction (Supplemental Figure 8b), we applied cell type label transfer analysis, where cell type annotations in scRNA-seq data were transferred to scATAC-seq data, to predict a corresponding cell type in the latter (Figure 7e-g). To do this, we annotated cells based on manual identification of the four scRNA-seq clusters, designating the two smaller activated HBC clusters “Activated_1_” and “Activated_2_” and the two larger HBC clusters “Activated” and “Resting.” (Figure 7e). Cell type transfer revealed that the “Activated_1_” and “Activated_2_” subclusters were recovered within the scATAC-seq hybrid cluster, and they were tightly compacted within the reduced-dimensional space (Figure 7f,g).

We then searched for marker genes and transcription factor motifs for each of the four new ATAC-seq clusters identified after label transfer and shown in Figure 7g (“Resting,” “Activated,” “Activated_1_,” and “Activated_2_”) using *ArchR* (see Methods) (Supplemental Figure 9a,b). Marker genes appeared similar between the “Activated_1_” and “Activated_2_” clusters, while the “Activated” and “Resting” clusters were readily distinguishable. Moreover, the top enriched transcription factor motifs were similar between “Activated_1_” and “Activated_2_” and corresponded to the motifs identified previously for the main hybrid cluster (shown in Figure 5d). The enrichment results again pointed to a heavy involvement of Fox TFs for the Activated_1_ and Activated_2_ ATAC-seq subclusters, which was supported by higher expression of the relevant Fox TFs (*Foxa1*, *Foxb1*, *Foxb2*, *Foxc1*, *Foxc2*, *Foxd1*, *Foxl1*, and *Foxs1*) in the “Activated_1_” and “Activated_2_” RNA-seq clusters (Supplemental Figure 9c,d).

Taken together, the rapid injury-induced upregulation of genes whose chromatin is accessible in a subset of uninjured cells (“hybrid” HBCs), suggests a latent activated or poised state in which some wound response genes are primed for rapid activation.

### Lineage-specific genes occupy open chromatin in a subset of quiescent and activated HBCs

**Figure 8:**
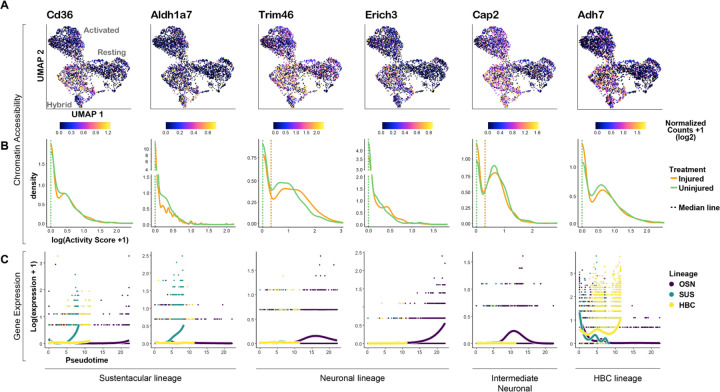
Lineage-specific genes are accessible in both uninjured and injured hybrid HBCs. **(A)** UMAP plots of normalized gene activity scores for lineage-specific genes in resting, hybrid, and activated HBCs. **(B)** Density plots of gene activity scores for each gene for hybrid cluster cells labeled by treatment. **(C)** Expression of each gene in each lineage over pseudotime.

Lineage-specific transcription appears primed in quiescent progenitor cells in several adult stem cell niches, including liver (Reizel et al. 2021; Iwafuchi-Doi et al. 2016), bone marrow (Paul et al. 2015), skeletal muscle (Okafor et al. 2023), and naive CD4+ T cells (Rogers et al. 2021). In order to investigate whether hybrid HBCs have accessible chromatin around lineage-specific genes, we ranked the marker genes for each ATAC-seq subcluster (identified above) by FDR (Supplemental File 2) and visualized high-ranking (low FDR) lineage-specific genes for any of the hybrid clusters by UMAP and density plots. Strikingly, we found that markers of Sus (*Cd36*, *Aldh1a7*), OSN (*Trim46*, *Erich3*, *Cap2*), and regenerated HBC (*Adh7*) lineages were enriched in the main hybrid cluster (Figure 8a). Importantly, we confirmed that the distribution of activity scores for each of these genes was comparable for injured and uninjured cells in the main hybrid cluster (Figure 8b, Supplemental Figure 10). Moreover, we confirmed lineage-enriched expression for each of these genes, as visualized using *tradeSeq* (Figure 8c). Together, these results suggest that a subset of silent lineage-specific genes occupy open chromatin in resting HBCs despite not being expressed until after HBC activation.

## Discussion

A stem cell’s epigenetic landscape contributes to its ability to respond to injury and regenerate discrete lineages; however, the specific gene regulatory pathways involved in triggering HBC activation and determining cell fate in the olfactory epithelium stem cell niche are still unclear. Here, we have used single-cell transcriptomic and epigenetic profiling of HBCs, the quiescent stem cell population of the olfactory epithelium, and their progeny to determine the transcription factor cascades that contribute to HBC differentiation into distinct lineages after acute injury. We also identified TF binding motifs in accessible chromatin that point to gene regulatory networks specific for resting and activated HBC states. In the process, we identified a subset of quiescent HBCs with an epigenetic profile similar to a subset of injured HBCs. Collectively called “hybrid” cells, these HBCs may represent a previously undetected HBC state defined by epigenetic priming -- perhaps through a mechanism involving Fox TFs -- of a subset of wound response and lineage-specific genes.

The “hybrid” HBC state may represent a previously noted yet underappreciated heterogeneity among quiescent and activated HBCs, although it is still unclear whether this heterogeneity reflects a functional difference, such as lineage restriction, between cells (Gadye et al., 2017). Recent studies have identified functional heterogeneity in other adult stem cell populations, including skeletal muscle stem cells (Der Vartanian et al. 2019; Scaramozza et al. 2019; Okafor et al. 2023), thymus epithelial progenitor cells (Rogers et al. 2021; Kadouri et al. 2020; Nusser et al. 2022), hair follicle stem cells (Ma et al. 2020; Yang et al. 2017; Joost et al. 2020), and hematopoietic stem cells (Paul et al. 2015; Perié et al. 2015; Rodriguez-Fraticelli et al. 2018). Recent advances in detecting such heterogeneity owe in part to advances in single-cell epigenetic profiling. Indeed, it has been suggested that early in differentiation, chromatin state may be better than gene expression at predicting a progenitor cell’s fate (Ma et al. 2020). It would therefore stand to reason that chromatin state may be a particularly good predictor of differences between seemingly homogeneous cells, and may explain why the hybrid HBC population was first detected at the epigenetic level, while the quiescent and activated HBC populations were observed to be two transcriptionally discrete cell states. It will be interesting in the future to determine whether their chromatin profiles of hybrid HBCs indicate prospective cell fate restrictions.

Whether or not hybrid HBCs are lineage restricted, the accessible chromatin around wound response genes, like *Emp1* and *Sprr1a*, in hybrid HBCs suggests that some quiescent HBCs are primed for activation at a subset of injury response genes in response to injury. The enrichment of Fox TF family motifs in hybrid ATAC-seq peaks is particularly interesting given our finding that FoxA1 is highly active early in the neuronal lineage, at the HBC* stage. FoxA TFs are established pioneer transcription factors that are able to overcome repressive chromatin states by displacing the linker histone and recruiting additional TFs and transcriptional cofactors (reviewed in (Iwafuchi-Doi and Zaret 2016)). Moreover, a recent study identified a small subset of primed pancreatic enhancers bound by FoxA TFs that are distinct from other pancreatic enhancers that recruit FoxA TFs only during lineage induction and have fewer and more degenerate FoxA motifs (Geusz et al. 2021). The strong enrichment of Fox motifs in hybrid HBCs could indicate transcriptional priming at a subset of hybrid-specific enhancers.

It has long been established that promoter co-occupancy of the Polycomb Repressive Complex 2 and the ESC pioneer TFs Oct4, Sox2, and Nanog in embryonic stem cells (ESCs) poises developmental genes for activation (Lee et al. 2006). Moreover, RNA polymerase II (Pol II) stalling at promoters of highly regulated genes is an established means of maintaining a gene’s potential to be reactivated (reviewed in (Core and Adelman 2019)). Here, we have identified a discrete population or state of HBCs that may be poised similarly -- perhaps via pioneer TFs at a subset of genes -- for activation of specific genes upon injury, thereby making a subset of quiescent and activated HBCs epigenetically indistinguishable. By contrast, for the majority of activated and quiescent HBCs, chromatin accessibility and TF motif enrichment was distinct, consistent with widespread differential gene expression between these two states. Indeed, while accessible chromatin in the resting HBC cluster was enriched for motifs for Trp63, a well established regulator of HBC quiescence, the accessible chromatin of injured HBCs was enriched for motifs for activity response proteins (c-Fos, Fosb, Junb, and Jund) and the main regulatory component of the BAF chromatin remodeling complex (Smarcc1), which was shown to regulate keratin gene expression in early embryonic stem cells, thereby promoting trophectoderm fate (Lim et al. 2020). Together, these genes represent the ongoing balance of quiescence versus proliferation in the olfactory epithelium stem cell niche. Given this ongoing balance, a third “hybrid” state that is poised for activation in response to injury may provide a strategy for reserving regenerative capacity while maintaining quiescence under homeostatic conditions.

## Methods

### Animals

All animal work was carried out in compliance with the University of California Institutional Animal Care and Use Committee (IACUC) according to federal guidelines. Mice containing the *Krt5-CreER(T2)* driver (Indra et al. 1999) and *Rosa26*^*eYFP*^ reporter (Srinivas et al. 2001) were kept on a mixed C57Bl/6 and 129 background. Both male and female mice were used in all studies.

### scRNA-seq with lineage tracing

#### OE injury, dissociation, and Fluorescence Activated Cell Sorting (FACS)

HBCs were labeled and their descendents post-injury were lineage traced using the *Krt5-CreER* driver crossed with the *Rosa26*^*eYFP*^ fluorescent reporter, as described previously ((Fletcher et al. 2017); (Gadye et al. 2017)). Briefly, *Krt5-CreER*; *Rosa26*^*eYFP/eYFP*^ mice were injected intraperitoneally once with tamoxifen (0.25 mg tamoxifen/g body weight) after weaning, then injected with methimazole (50 μg/g body weight, IP) at least one day after tamoxifen administration, and sacrificed at 24 h, 48 h, 96 h, 7 d, or 14 d after injury with methimazole. For each experimental time point, the dorsal olfactory epithelium (OE) was surgically removed and dissociated, as described in Fletcher et al. 2017 and Gadye et al. 2017: OE from each animal was individually processed in approximately 1 mL of pre-warmed (37°C) dissociation medium (150 units papain dissolved in 5 mL Neurobasal medium with 2.5 mM Cysteine and 2.5 mM ethylenediaminetetraacetic acid) with 100 units DNAse I and incubated at 37°C for 25 mins. Samples were then washed three times with 10% fetal bovine serum in phosphate buffered saline (PBS-FBS) and strained through a 35 μm nylon mesh filter cap into a 5 mL polypropylene tube to remove debris. Propidium iodide (PI) was added to cells at a final concentration of 2 μg/mL just before loading them onto a BD Influx cell sorter. After running negative controls (no YFP, no PI and no YFP, PI only), YFP-positive/PI-negative cells were collected in a low-binding 1.5 mL tube containing 10% FBS in PBS on ice.

Each FACS collection was considered a biological replicate. When possible, at least one male and one female mouse was used per biological replicate to aid doublet identification, and a minimum of two biological replicates were collected per condition. For each replicate, age-matched animals were given the same treatment (See Table 1 for a summary of the experimental design for each sequencing modality in this study).

#### Cell capture and single-cell RNA sequencing

The 10x Genomics droplet-based transcriptome profiling system (Zheng et al Nat Comm 2017) was used to capture single cells, lyse them, and produce cDNA. Reverse transcription (RT) mix was added to the single-cell suspension and loaded onto the Single Cell B Chip. The Chromium Single Cell 3’ GEM, Library, and Gel Bead Kit, Chromium Chip B Single Cell Kit, and Chromium i7 Multiplex Kit were used for RT, cDNA amplification, and library preparation according to the manufacturer’s instructions (Chromium Single Cell 3’ Reagents Kits v2). Indexed single-cell libraries were sequenced in multiplex on Illumina HiSeq 4000 sequencers to produce 100bp paired-end reads.

### scRNA-seq data analysis

#### Initial processing, normalization, and clustering

Fastq files were generated from binary base call files, aligned to *mm10*, quantified, and aggregated using *Cell Ranger* v2.0.0 to produce a feature-barcode matrix containing the number of unique molecular identifiers (UMIs corresponding to cells) associated with a feature corresponding to a gene (row) and a barcode corresponding to a biological sample (column), and a molecule information file containing the number of reads assigned with high confidence to a gene for each UMI. Initial preprocessing of the molecule information file was performed as described in Brann *et al.* (2020). Afterwards, for each UMI (cell), the gene expression data were scaled by the median total counts across all cells. Dimensionality reduction was performed using principal component analysis (PCA) and the top 20 principal components were used as input to UMAP (McInnes, Healy, and Melville 2018). Clustering was performed using the *SCANPY* toolkit (Wolf, Angerer, and Theis 2018) using the Leiden algorithm with resolution parameter equal to 1.45, and a manual merging of clusters was performed using known marker genes. We identified 5,418 activated HBCs (HBC*), 7,782 regenerated HBCs (rHBC), 755 globose basal cells (GBC), 2,683 sustentacular cells (Sus), 2,859 immature olfactory sensory neurons (iOSN), and 929 mature olfactory sensory neurons (mOSN). A group of 176 microvillous cells as well as 964 respiratory cells were removed as they did not belong to the trajectory.

#### Trajectory inference

The 1,000 most variable genes were selected for dimensionality reduction prior to trajectory inference. PCA on log-transformed counts was performed, calculating the top 25 PCs using the R package *irlba*, which were subsequently reduced to three dimensions using UMAP with parameters min_dist=0.2, n_neighbors=15. Hierarchical clustering based on Euclidean distance in the 3D UMAP-space was performed. The hierarchical tree was cut to obtain nine clusters, which were used as input to *slingshot* for trajectory inference, setting the starting cluster to correspond with HBC* cells.

#### Differential gene expression analysis

Negative binomial generalized additive models (NB-GAM) were fitted using *tradeSeq*. The number of knots for each lineage was set to 6 based on the Akaike Information Criterion (AIC). Top upregulated genes for each lineage, as shown in Figure 1, were identified using the associationTest implemented in *tradeSeq*, requiring a gene to be significant at a 5% nominal FDR level, as well as having a higher estimated average gene expression at the end of the lineage as compared to the lineage starting point based on the NB-GAM.

#### Transcription factor cascade

A list of transcription factors was obtained from the Animal Transcription Factor Database as described previously (Fletcher et al. 2017) and can be found at https://github.com/rufletch/p63-HBC-diff.

We implemented new functionality in tradeSeq to calculate first derivatives of the smooth fitted gene expression functions from the NB-GAM, using finite differencing. Letting $Y_{gi}$ be the gene expression measure for gene g in cell i, the tradeSeq model is defined as

$$Y_{gi}\sim NB\left(\mu_{gi},\phi_{g}\right)$$

$$\log\left(\mu_{gi}\right)=\eta_{gi}$$

$$\eta_{gi}=\sum_{l=1}^{L}s_{gl}\left(T_{li}\right)Z_{li}+{\text{U}}_{i}\alpha_{g}+\log\left(N_{i}\right),$$

where the mean $\mu_{gi}$ of the negative binomial (NB) distribution is linked to the additive predictor $\eta_{gi}$ using a logarithmic link function.

The gene-wise additive predictor consists of lineage-specific smoothing splines $s_{gl}$, that are functions of pseudotimes $T_{li}$, for lineages $l\in \left\{1,\ldots ,L\right\}$. The binary matrix $\text{Z}=\left(Z_{li}\in \left\{0,1\right\}:l\in \left\{1,\ldots ,L\right\},i\in \left\{1,\ldots ,n\right\}\right)$ assigns every cell to a particular lineage based on user-supplied weights. The n×p matrix U is a model matrix allowing the inclusion of p known cell-level covariates (e.g., batch, age, or gender), with $i^{\text{th}}$ row $U_{i}$ corresponding to the $i^{\text{th}}$ cell; $\alpha_{g}$ is a regression parameter vector of dimension p×1.

Differences in sequencing depth or capture efficiency between cells are accounted for by cell-specific offsets $N_{i}$.

The smoothing spline $s_{gl}$, for a given gene g and lineage l, can be represented as a linear combination of K cubic basis functions,

$$s_{gl}\left(t\right)=\sum_{k=1}^{K}b_{k}\left(t\right)\beta_{glk},$$

where the cubic basis functions $b_{k}\left(t\right)$ are enforced to be the same for all genes and lineages. Here, we use six knots, thus, for each gene and each lineage in the trajectory, we estimate K=6 regression coefficients $\beta_{glk}$.

#### Estimation of first derivatives.

Derivatives of GAMs can be approximated using finite differencing (Wood, 2017). Specifically, assuming a vector of J pseudotime grid points ${\text{t}}_{l}^{*}=\left(t_{lj}^{*}:j\in \left\{1\ldots ,J\right\}\right)$ for each lineage l, then the derivatives of the splines $s_{gl}$ at these grid points are approximated by

$${\hat{\delta }}_{glj}=\frac{\partial }{\partial t}s_{gl}\left(t_{lj}^{*}\right)\approx \sum_{k=1}^{K}\left[\frac{b_{k}\left(t_{lj}^{*}+\epsilon \right)-b_{k}\left(t_{lj}^{*}-\epsilon \right)}{2\epsilon }\right]{\hat{\beta }}_{glk},$$

where ϵ>0 corresponds to a small finite number, here taken to be $10^{-7}$.

Standard errors ${\hat{\sigma }}_{{\hat{\delta }}_{glj}}$ on ${\hat{\delta }}_{glj}$ are similarly obtained (Ruppert et al. (2003), Wood (2017)), since the derivatives are a linear combination of the smoother coefficients $\beta_{glk}$.

In our application, derivatives for each gene are calculated over a grid of J=100 equally-spaced points for each lineage and, for each grid point, we calculate a thresholded test statistic

$$T_{glj}=\frac{\max\left(0,{\hat{\delta }}_{glj}-c\right)}{{\hat{\sigma }}_{{\hat{\delta }}_{glj}}},$$

where we set the threshold c to be equal to 0.1.

For each gene separately, we considered whether the first derivative of the NB-GAM fit was significantly different from zero for at least one of 100 grid points. Specifically, one-sided *p*-values for the test of the null hypothesis that the derivative is greater than an arbitrary threshold of 0.1 were calculated at each grid point using a standard normal null distribution. Next, Holm (1979) adjusted *p*-values were computed across grid points and TFs with adjusted *p*-value below a 5% cut-off were declared involved in the cascade. The expression peak of each involved TF was defined as the point where the first derivative crosses zero after the grid point corresponding to the lowest adjusted *p*-value. Note that we merely view the *p*-values as useful numerical summary statistics, without attaching strong probabilistic interpretations to them.

#### Identifying TFs most active in a particular lineage

To identify TFs that are most active in one lineage as compared to other lineages, we start from the set of TFs found to be involved in each lineage from the procedure described above. Once a TF is included in that set, we consider it to be most active in a particular lineage if the maximum of the estimated lineage-specific smoother is at least 1.5 times larger in that respective lineage as compared to the other two lineages. The 50% increase threshold was chosen to represent a biologically meaningful difference between lineages.

#### Identifying shared TFs across lineages

To identify TFs that are involved across all three lineages, we start from the set of TFs found to be involved in each lineage from the procedure described above. We consider a TF to be ‘shared’, if its expression peak is at a similar pseudotime for all lineages. Out of 90 TFs that peak in all three lineages, we consider 19 TFs to be shared, as the pseudotime difference of their expression peak is lower than 1.

#### Clustering and gene set enrichment analysis

Log-transformed and scaled transcription factor expression measures for all TFs found to be involved in each lineage were clustered using hierarchical clustering based on Euclidean distance, and the hierarchical tree was cut at a predefined number of clusters. We somewhat arbitrarily chose 4 clusters for the neuronal lineage and 3 clusters for the sustentacular and rHBC lineages, as more TFs are involved in the neuronal lineage. Gene set enrichment analysis was performed using hypergeometric tests based on the TFs of each cluster using hallmark gene sets from the MSigDB database (Liberzon et al. 2015). For each group, we considered the top 10 enriched gene sets for interpretation purposes.

#### Deconvolution of gene expression to TF activity

For transcription factor activity analysis we only focused on the cells that were assigned to the neuronal lineage, based on the cell assignment procedure from *tradeSeq* and the trajectory fitted using *slingshot* (Street *et al.* 2018, Van den Berge *et al.* 2020). After subsetting, the dataset consists of 14,618 genes and 6,810 cells. The gene regulatory network (GRN) was estimated using grnboost2, implemented in SCENIC v0.10.2 (Aibar *et al.* 2017). The estimated GRN contains 7,863 genes, 262 transcription factors, and 25,896 edges. The median number of genes regulated by a TF is 27. Transcription factor activity was estimated using *transfactor* (manuscript in preparation), a statistical method which leverages a GRN to assign mRNA molecules to the transcription factors that produced them. The number of molecules produced by each TF in each single cell is then used as a proxy for its activity. Specifically, *transfactor* relies on a hierarchical Poisson model for the number of transcripts produced by each TF for a given gene. The EM algorithm is then used to fit the model and deconvolve TF-specific gene expression from overall gene expression for each gene.

### Bulk ATAC-seq

#### OE injury, dissociation, and FACS

Injury, removal, and dissociation of the OE was performed as described above with the following changes: wild-type CD1 mice 6–8 weeks-old were used, and only two conditions were assessed -- UI and 24 HPI (3 biological replicates, each consisting of a mix of cells from 2–3 mice, per condition). After papain incubation and 3 washes with 10%FBS in PBS (PBS-FBS), anti-ICAM1-PE antibody was added to the cell suspension to a final concentration of 10 μg/mL and incubated at 4°C for 20 minutes protected from light. Cells were then washed once with ice cold PBS-FBS, resuspended in cold PBS-FBS, and strained through a 35 μm nylon mesh filter cap into a 5 mL polypropylene tube to remove debris. Propidium iodide (PI) was added to cells at a final concentration of 2 μg/mL just before loading them onto a BD inFlux or BD FACSAria cell sorter. After running negative controls (no antibody, no PI and no antibody, PI only), ICAM1-PE-positive/PI-negative cells were collected in a low-binding 1.5 mL tube containing PBS-FBS on ice.

#### Library construction and sequencing

ATAC-seq libraries were generated from FACS-purified ICAM1-PE-positive cells as described in (Corces et al. 2017). Briefly, 10,000–15,000 cells were collected in 100 μl cold PBS-FBS, pelleted at 500 RCF for 5 min at 4°C, and lysed on ice for 3 mins in 50 μl cold lysis buffer (1% Digitonin, 10% Tween-20, 10% NP40 in 1M Tris-HCl pH 7.4, 5M NaCl, 1M MgCl_2_). Cold wash buffer (10% Tween-20 in 1M Tris-HCl pH 7.4, 5M NaCl, 1M MgCl_2_) was added, and tubes were inverted 3 times. Nuclei were then pelleted at 500 RCF for 10 mins at 4°C, supernatant was removed, and 20 μl transposition mix (1X Tagment Buffer, 0.01% Digitonin, 0.1% Tween-20, 1 μl TDE1) was added to each sample. Nuclei in transposition mix were incubated at 37°C for 30 minutes in a thermomixer with 1000 RPM mixing. Immediately after transposition, samples were placed on ice and 5 volumes of DNA Binding Buffer (Zymo) were added to each. Reactions were cleaned up using a Zymo DNA Clean and Concentrator 5 kit, and samples were eluted in 20 μl Elution Buffer (Zymo).

For library generation, 18 μl of each sample was used as template for 5 cycles of PCR, then qPCR was performed on 4 μl of PCR product to assess the appropriate number of additional PCR cycles. The remaining 36 μl PCR reaction was run for the determined number of cycles. AMPure XP beads were used to select fragments between 180 and 1130bp, DNA concentration was measured using a Qubit dsDNA HS kit, and library quality was visualized using a Bioanalyzer. If fragments less than 100bp were observed, the library was further cleaned up using a Zymo DNA Clean and Concentrator 5 kit and eluted with 10 μl of 10 mM Tris-HCl, pH 8.0. Each replicate per condition was sequenced on a HiSeq 4000 (Illumina) in a different batch such that each sequencing batch contained one replicate from each condition.

### Bulk ATAC-seq data analysis

#### Peak calling

Adapters were trimmed using Trimmomatic (Bolger, Lohse, and Usadel 2014), and reads were aligned to mm10 with bowtie2 (Langmead and Salzberg 2012). After removing reads mapped to the mitochondrial genome, duplicated reads, and reads mapping to any region with fewer than 10 reads with SAMtools (Danecek et al. 2021), the remaining reads were used for peak calling with MACS2 (--shift −50 --extsize 100) (Zhang et al. 2008) and Genrich (https://github.com/jsh58/Genrich).

#### Differential accessibility

Within each condition, replicate peaks identified using each tool (MACS2 or Genrich) were intersected using bedtools *intersect* (Quinlan and Hall 2010), then peaks identified using MACS2 were intersected with peaks identified using Genrich using bedtools *intersect*. 65,998 total peaks in all samples were quantified using bedtools *merge*. Peaks were then annotated and ATAC-seq signals were quantified and normalized to library size, peak width, and GC-content using *cqn* (Hansen et al. 2012). Differentially accessible (DA) peaks between UI HBCs and 24HPI HBCs were identified using *edgeR* (Robinson, McCarthy, and Smyth 2010) for a total of 15,815 DA peaks.

### Bulk RNA-seq

#### OE injury, dissociation, and FACS

Injury, removal, and dissociation of the OE was performed as for bulk ATAC-seq, for a total of 2 biological replicates per condition. Each biological replicate consisted of a mix of cells from 2 male and 2 female age-matched mice.

#### Library construction and sequencing

RNA-seq libraries were generated from 20,000–50,000 FACS-purified ICAM1-PE-positive cells collected in 100 μl cold PBS-FBS, pelleted at 500 RCF for 5 min at 4°C, and resuspended in 100 μl TRI Reagent (Zymo). Total RNA was extracted using a Zymo Direct-zol RNA MicroPrep kit, rRNA was depleted using a NEBNext rRNA Depletion Kit (New England BioLabs Cat# E6310), and libraries were made using a NEBNext Ultra II Directional RNA Library Prep Kit for Illumina (New England BioLabs Cat# E7760). All four samples (two biological replicates from two conditions) were sequenced together in one run on a HiSeq 4000 (Illumina).

#### Bulk RNA-seq data analysis

Sequencing reads were mapped to *mm10* using *STAR* v 2.7.1a (Dobin et al. 2013). After removal of duplicated and low-quality reads using SAMtools (Danecek et al. 2021), reads were quantified using HOMER (Heinz et al. 2010). Differential expression was determined using *DESeq2* (Love, Huber, and Anders 2014).

### scATAC-seq with lineage tracing

#### OE dissociation and FACS Purification

Injury, removal, and dissociation of the OE were performed as described above with the following changes: *Krt5-CreER*; *Rosa26*^*eYFP/eYFP*^ mice were injected intraperitoneally once with tamoxifen (0.25 mg tamoxifen/g body weight) at 68 weeks of age, injected with methimazole (50 μg/g body weight, IP) or saline 3 days after tamoxifen administration, and sacrificed 24 h after injury (3 biological replicates per condition, and each replicate consisted of 2–3 mice.

#### Nuclei Isolation

Viable YFP+ cells (range: 900–3600) were sorted into 200 μl of PBS-FBS using a FACSAria (BD), centrifuged at 500g for 5 min at 4°C. After supernatant removal, cells were resuspended in 200 μl 0.04% BSA in PBS and centrifuged again at 500g for 5 min at 4°C. 195 μl supernatant was removed, and 45 μl cold lysis buffer (10mM Tris-HCl pH 7.4, 10mM NaCl, 3 mM MgCl2, 0.1% Tween-20, 0.1% NP40, 0.01% Digitonin, 1% BSA) was added and mixed with the pellet by gently pipetting up and down 3 times. Samples were incubated on ice for 3 min, and 50 μl cold wash buffer (10mM Tris-HCl pH 7.4, 10mM NaCl, 3mM MgCl2, 0.1% Tween-20, 1% BSA) was added to each without mixing. Samples were centrifuged at 500g for 5 min at 4°C. 95 ul supernatant was carefully removed, 45ul cold 1X Nuclei Buffer was added to each sample without mixing, and samples were centrifuged again at 500g for 5 min at 4°C. 48 μl supernatant was removed without disturbing the pellet, which was then resuspended in the remaining (~5 μl ) buffer for use in single cell ATACseq.

#### 10x Genomics ATAC-seq library construction

Nuclei isolated as described above were used for preparing single-cell ATAC-seq libraries using a Chromium Single Cell ATAC Library & Gel Bead Kit (PN-1000111) according to the manufacturer’s instructions (10x Chromium Single Cell ATAC Reagents Kits User Guide). Briefly, 5 μl of each sample resuspended in 1X Nuclei Buffer was added to 10 μl Transposition Mix, mixed by pipetting 6 times, and incubated in a thermal cycler at 37°C for 1 hour. After addition of barcoding master mix, the Chromium Chip E Single Cell Kit (PN-1000086) and Chromium i7 Multiplex Kit N, Set A (PN-1000084) were used for GEM generation and library construction. Indexed single-cell libraries were sequenced in multiplex on a NovaSeq (Illumina) to produce 150bp paired-end reads.

### scATAC-seq data analysis

Raw data were processed using the *Cell Ranger* scATAC-seq processing pipeline. Downstream analysis was performed using *ArchR* (Granja et al. 2021) v0.9.5, discarding cells that had fewer than 1,000 fragments or a transcription start site (TSS) enrichment score less than 4. Doublets were removed by *ArchR* using default settings. Gene activity scores were calculated using default settings in *ArchR*. Latent semantic indexing was performed to obtain 30 reduced dimensions, which were subsequently corrected for sample/batch effects using *Harmony* (Korsunsky et al. 2019). Clustering was performed on the *Harmony* embeddings using the ‘Seurat’ method implemented in *ArchR*, obtaining 10 clusters which were merged manually. For visualization purposes, the Harmony embeddings were used as input to UMAP (McInnes, Healy, and Melville 2018) to reduce it to two dimensions. Gene markers for the HBC cell states were obtained based on the estimated gene activity scores using the getMarkers implementation in *ArchR*, using a Wilcoxon test with a log-fold-change cut-off of 1.25 and Benjamini-Hochberg FDR nominal level of 1% as thresholds.

Pseudobulking, where counts are summed across cells that share the same cell state/type, was performed using the addGroupCoverages implementation in *ArchR*, creating a minimum of 2 and maximum of 6 pseudobulks for each of the three HBC cell states. Peak calling was performed using *MACS2* (Zhang et al. 2008) on the pseudobulks, resulting in 183,712 peaks. Peak markers for the HBC cell states were obtained as described in the previous paragraph, using a Wilcoxon test statistic with a log-fold-change cut-off of 1.25 and Benjamini-Hochberg FDR nominal level of 1% as thresholds. Motif enrichment as implemented in *ArchR* (Granja et al. 2021) for the marker peaks of each cell state was performed using the cisbp database (Weirauch et al. 2014).

### Integration of scRNA-seq and scATAC-seq data

We used *Seurat* v3.1.5 for integration of the scRNA-seq and scATAC-seq datasets (Stuart et al. 2019). For the scATAC-seq data, we used the peak matrix and gene activity matrix obtained using *ArchR* (Granja et al. 2021) as described in the previous section. Integration was performed by identifying anchor cells across datasets using canonical correlation analysis on the scRNA-seq gene expression and scATAC-seq gene activity scores. Cell labels were transferred from the scRNA-seq to scATAC-seq data using these anchor cells, as implemented in *Seurat*’s TransferData function. Marker peaks/genes and motif enrichment was performed for each cell identity using *ArchR* as described in the previous section.

## Supplementary Material

Supplement 1

Supplement 2

Supplement 3

## Figures and Tables

**Table 1:** Summary of purification strategies, conditions, biological replicates, and age ranges of animals used in this study for each sequencing modality.

Modality	FACS Purification Strategy	Conditions	Biological replicates per condition	Mice pooled per bio. replicate	Age range
scRNA-seq	Krt5CreER; R26-YFP lineage traced cells	24, 48, 96 HPI 7, 14 DPI	2	1–6	3–7 weeks
bulk RNA-seq	ICAM+ HBCs	UI, 24 HPI	2	4 (2M, 2F)	6.1–8 weeks
scATAC-seq	Krt5CreER; R26-YFP lineage traced cells	UI, 24 HPI	3	3	5.6–7.9 weeks
bulk ATAC-seq	ICAM+ HBCs	UI, 24 HPI	4	2–3	6–8 weeks
