# Supplementary material for: A Latent Activated Olfactory Stem Cell State Revealed by Single Cell Transcriptomic and Epigenomic Profiling
Source: bioRxiv. 2023 Oct 31:2023.10.26.564041. Preprint. [Version 1] doi: 10.1101/2023.10.26.564041 (PMC10634988; doi:10.1101/2023.10.26.564041)
Supplement: Supplementary file 1 [file media-3.pdf]

Supplemental Figures for Van den Berge et al.

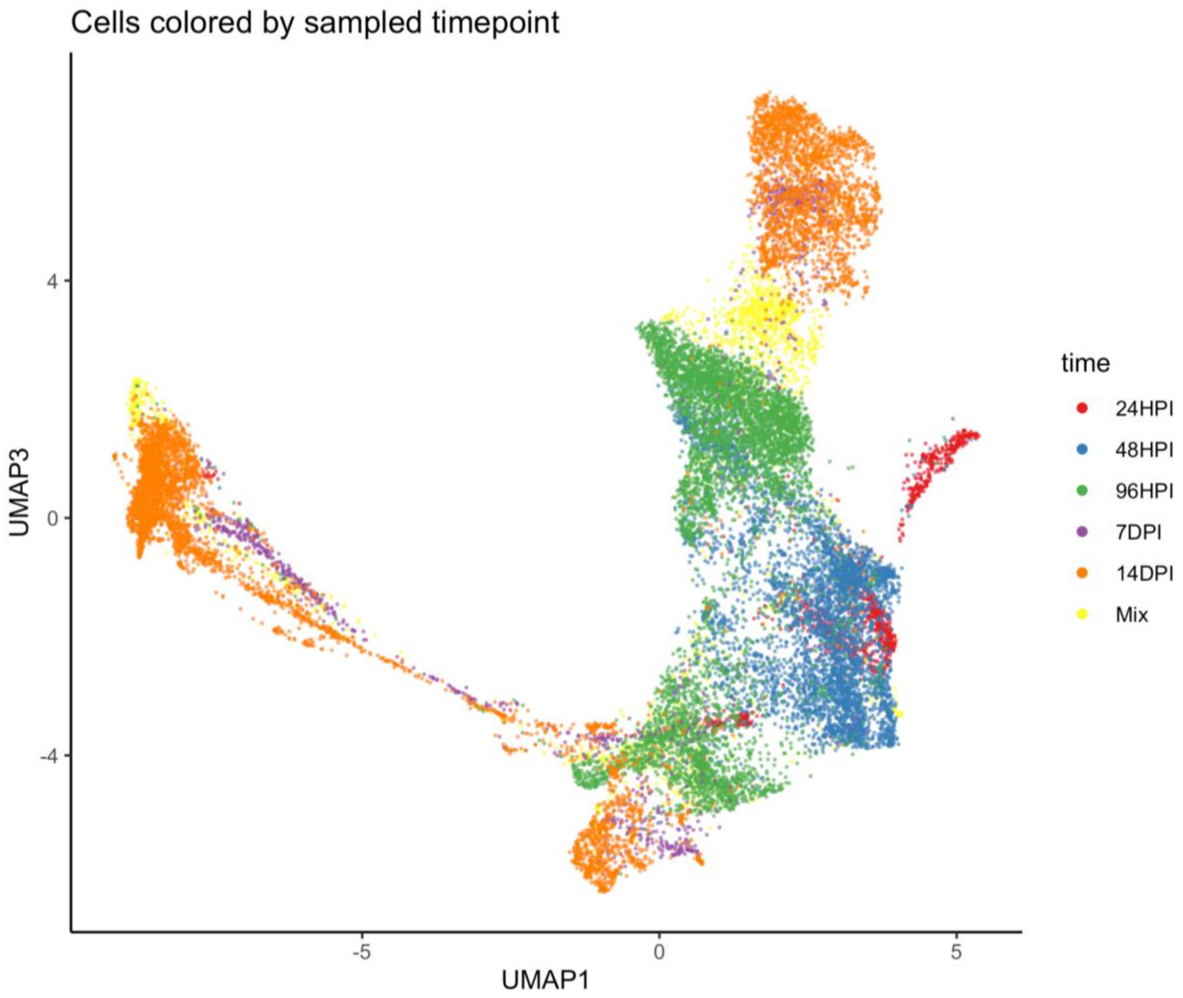

Supplemental Figure 1: UMAP dimensionality reduction of the cells colored according to their experimentally sampled timepoint. For improved visualization, UMAP dimensions 1 and 3 are visualized.

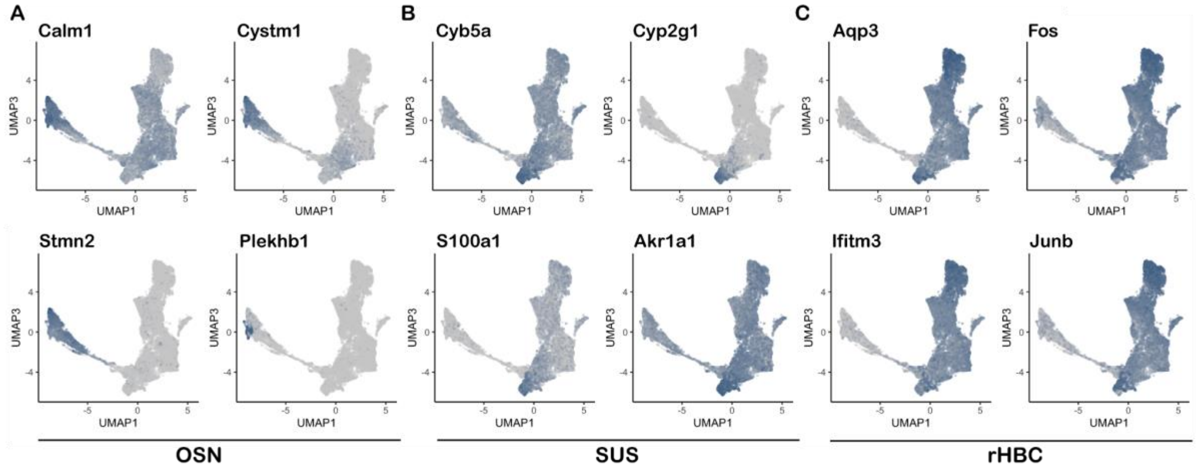

Supplemental Figure 2: Markers identified by tradeSeq in 2D UMAP space for the (A) neuronal lineage, (B) sustentaclular lineage, and (C) rHBC lineage. Darker color corresponds with higher expression.

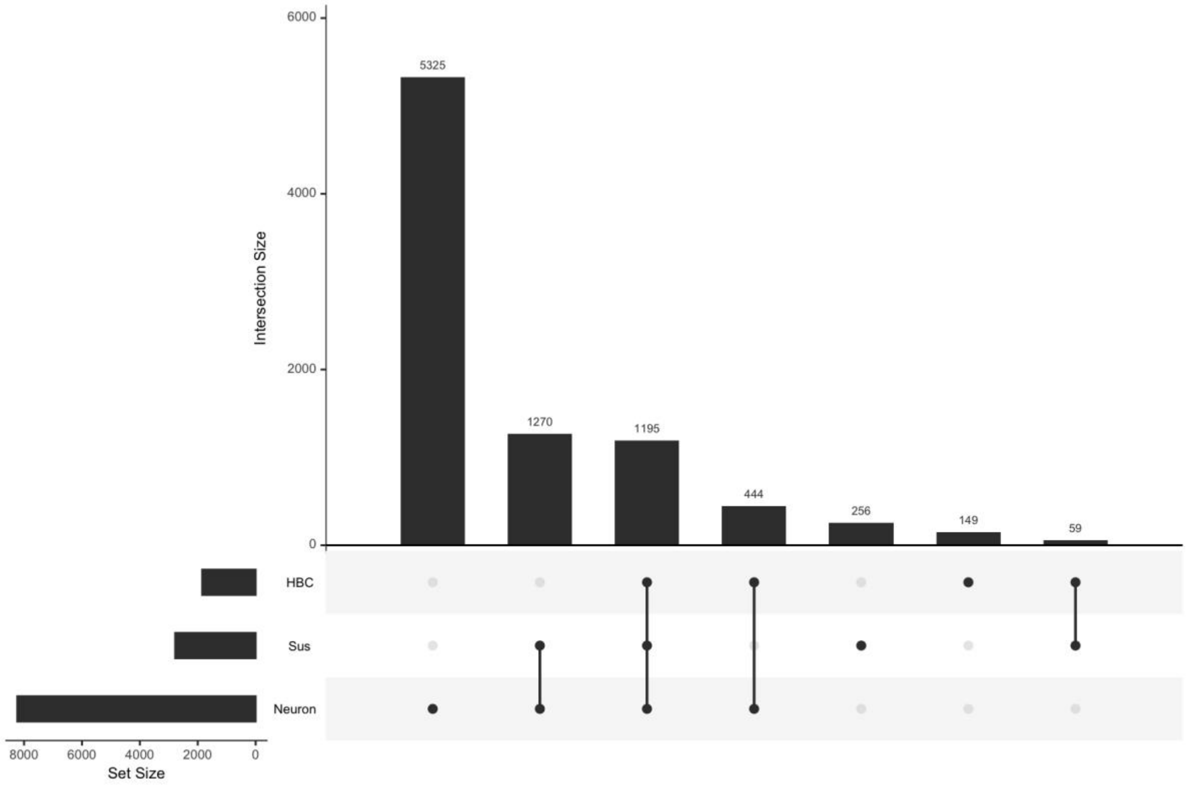

Supplemental Figure 3: UpSet plot comparing overlap of differentially expressed genes for each lineage. Left panel: Barplot of the number of DE genes for each lineage on a 5% nominal FDR level. Top panel: Barplot of the number of DA peaks in common for every combination of lineages. Center panel: Combination of methods under consideration for the top panel. For example, the first column shows that 5,325 DE genes are only found to be associated with pseudotime in the neuronal lineage.

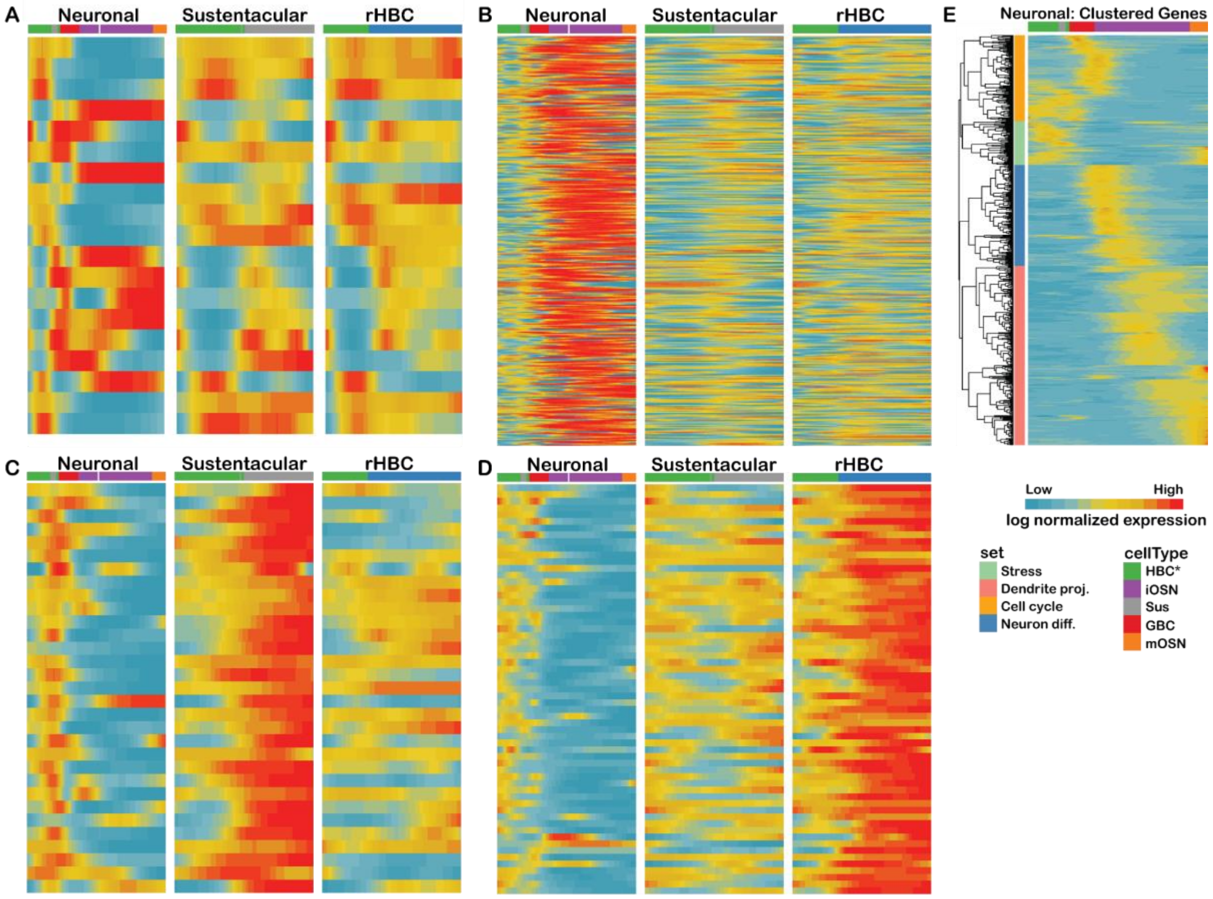

Supplemental Figure 4: Interpretation of TF activity cascades. ***(A-D)*** *Heatmaps for TFs found to be shared across all lineages (A), most active in the neuronal lineage (B), most active in the sustentacular lineage (C), and most active in the regenerated HBC lineage (D). The x-axis for each panel represents 100 bins of pseudotime, and the most abundant cell type in each bin is indicated using the colors at the top of each heatmap. If there are too few cells in a bin, no color is provided. Each row in each heatmap represents scaled log-transformed expression of a transcription factor, normalized to zero mean and unit variance across all lineages. The list of shared TFs is available in Supplemental File 1.* ***(E)*** *Clustered heatmap of scaled log-normalized expression for TFs (rows) involved in the neuronal lineage. The x-axis represents pseudotime and the dominant cell type in each pseudotime bin is indicated at the top of the heatmap. The TFs are clustered into four groups using hierarchical clustering. For each cluster, gene set enrichment analysis is performed; enriched gene sets correspond to known biology.*

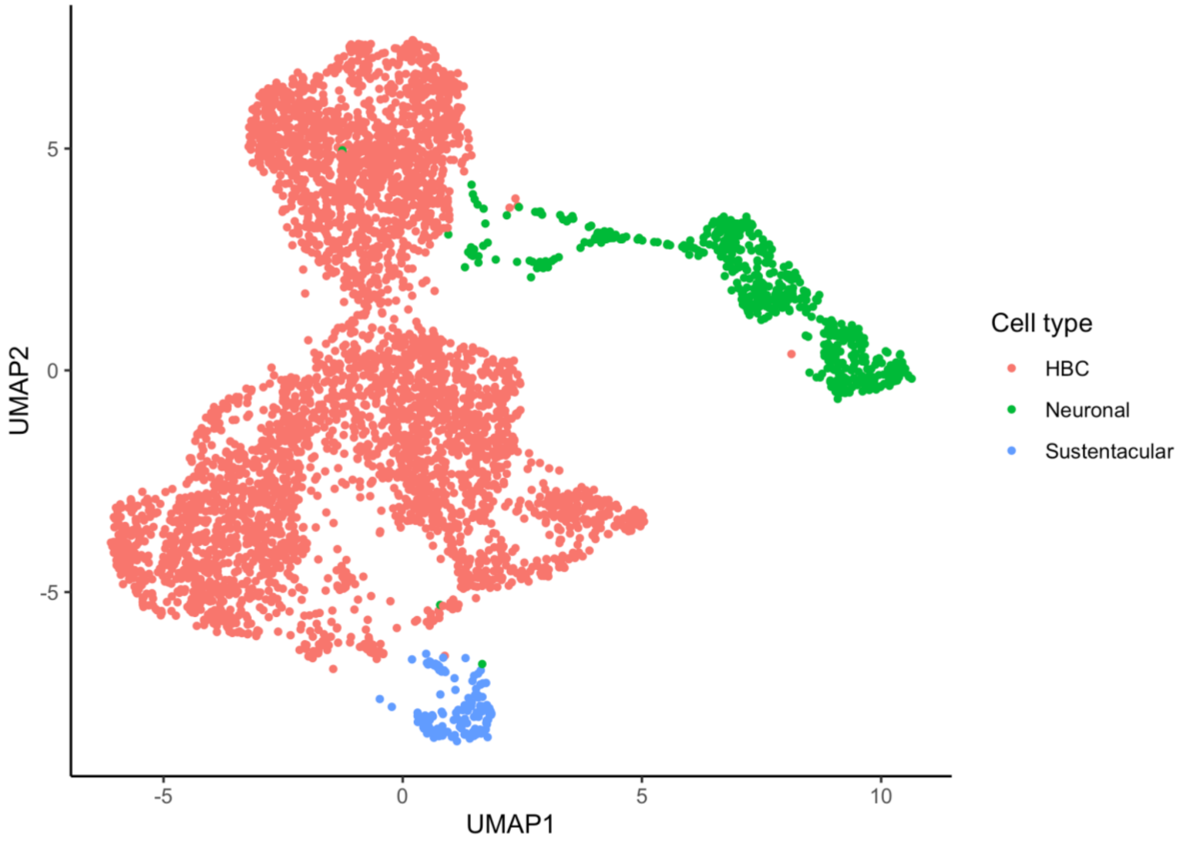

Supplemental Figure 5: UMAP dimensionality reduction of all scATAC-seq cells after quality control. Each cell is colored according to its cell type, as defined by manually merged clusters, with the majority of cells corresponding with the HBC cell type, and two smaller clusters corresponding with neuronal and sustentacular cells.

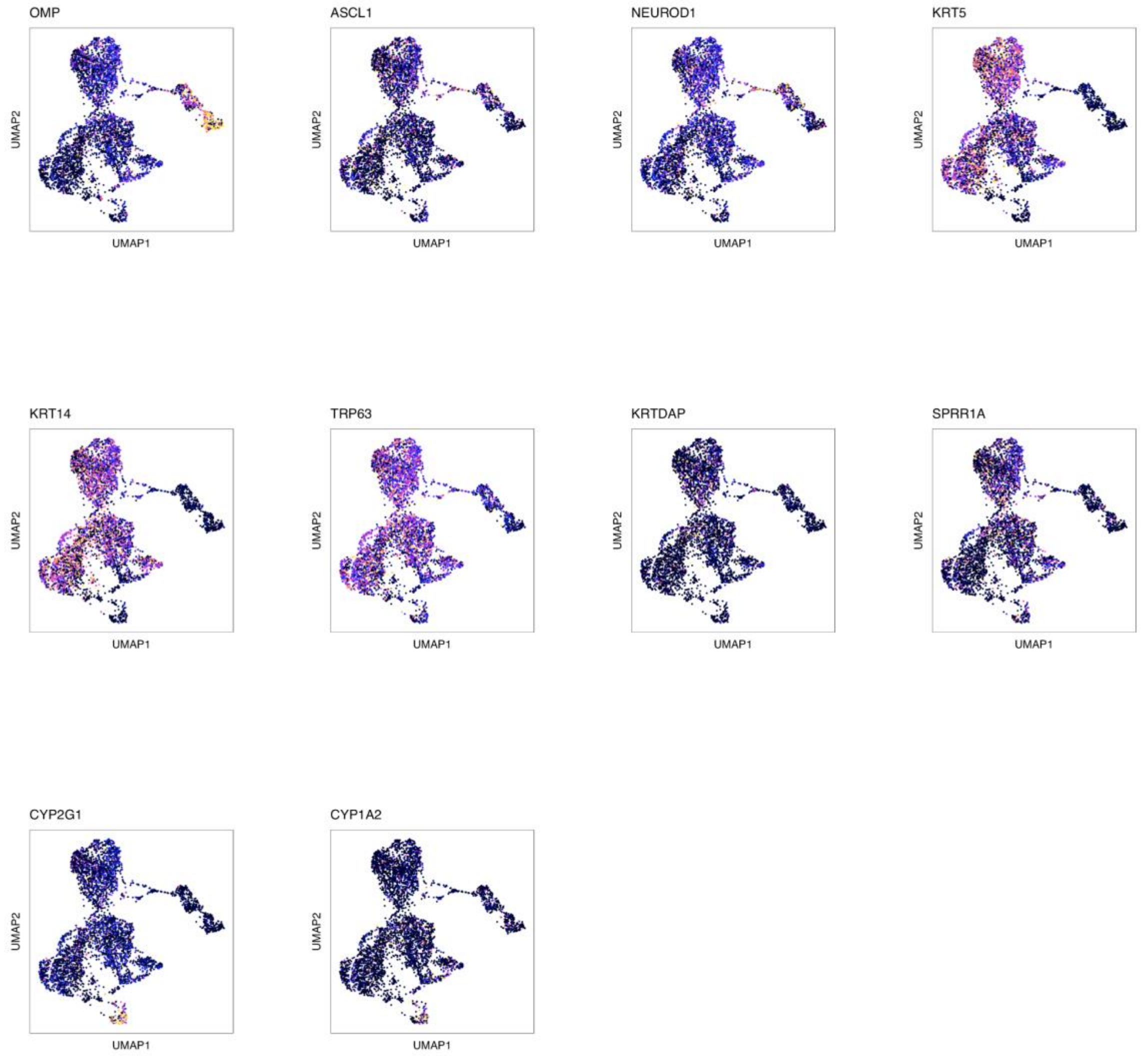

Supplemental Figure 6: scATAC-seq data in 2D UMAP space, colored by known markers. Each panel shows a two-dimensional UMAP visualization of the scATAC-seq cells. Cells are colored according to the estimated gene activity of the corresponding marker gene as indicated by the panel title. Lighter color/yellow corresponds with higher gene activity.

A

	Activated		Hybrid		Resting	
**Gene**	**Median**	**Mean**	**Median**	**Mean**	**Median**	**Mean**
**Icam1**	0	0.36	0.66	0.67	0.82	0.79
**Krt5**	1.02	0.95	0.43	0.55	0.78	0.75
**Krt6a**	0.34	0.53	0	0.18	0	0.15
**Ecm1**	0.57	0.60	0.16	0.33	0	0.19
**Sprr1a**	0	0.36	0	0.33	0	0.17
**Emp1**	0.94	0.92	1.00	0.98	0.39	0.39

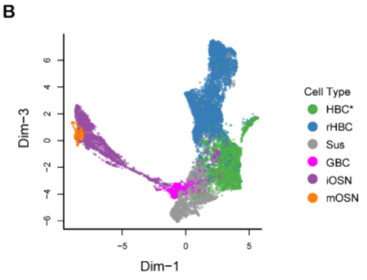

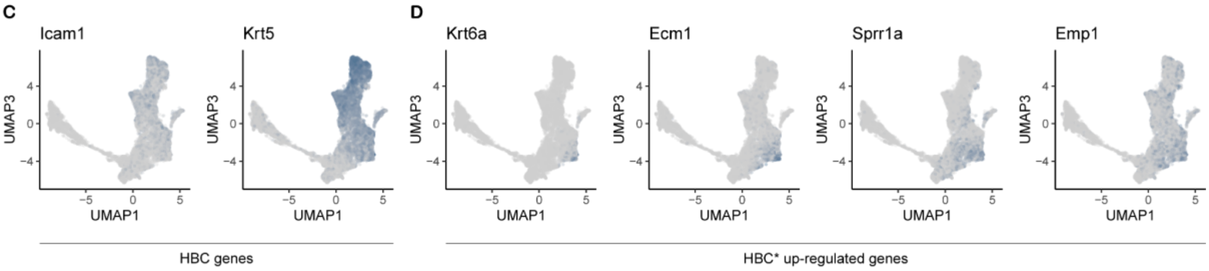

Supplemental Figure 7: Some wound response genes have similar gene activity in the hybrid and activated states compared with the resting state. ***(A)*** *Median and mean values of wound response gene activity scores for cells in the scATAC-seq activated, hybrid, or resting clusters.* ***(B)*** *Legend showing cell type clusters by 2D UMAP of scRNA-seq data from lineage traced cells.* ***(C,D)*** *Scaled expression of HBC marker genes (c) and wound response genes (d) visualized by 2D UMAP. Darker color corresponds with higher expression. Wound response genes are enriched in the HBC* cluster.*

A

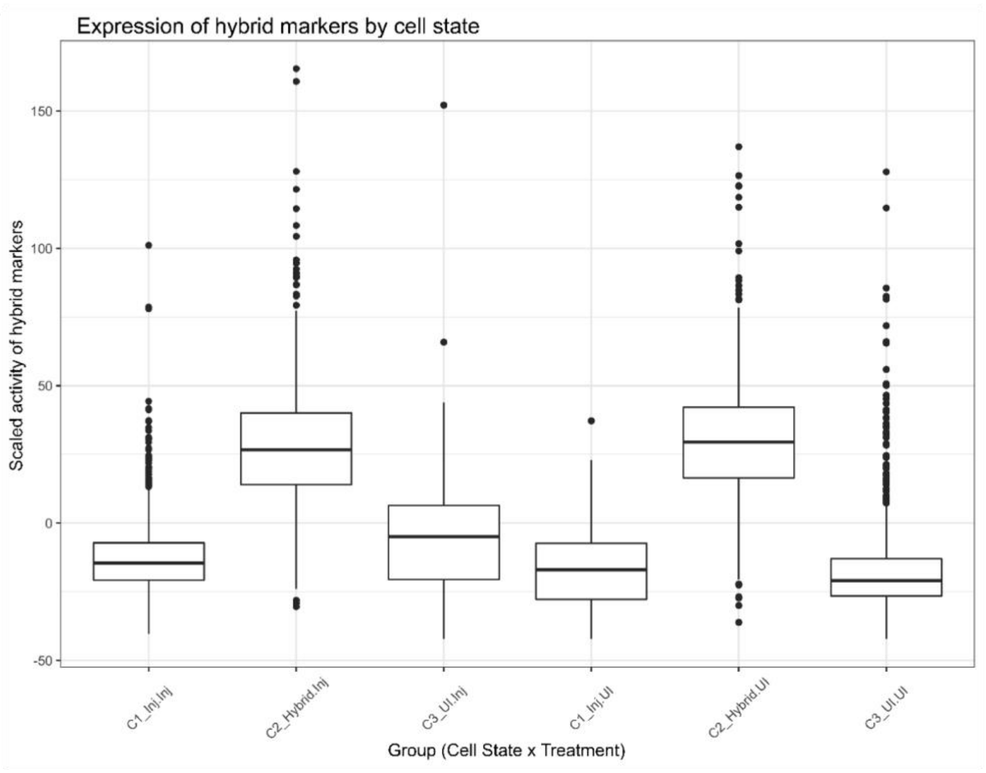

B

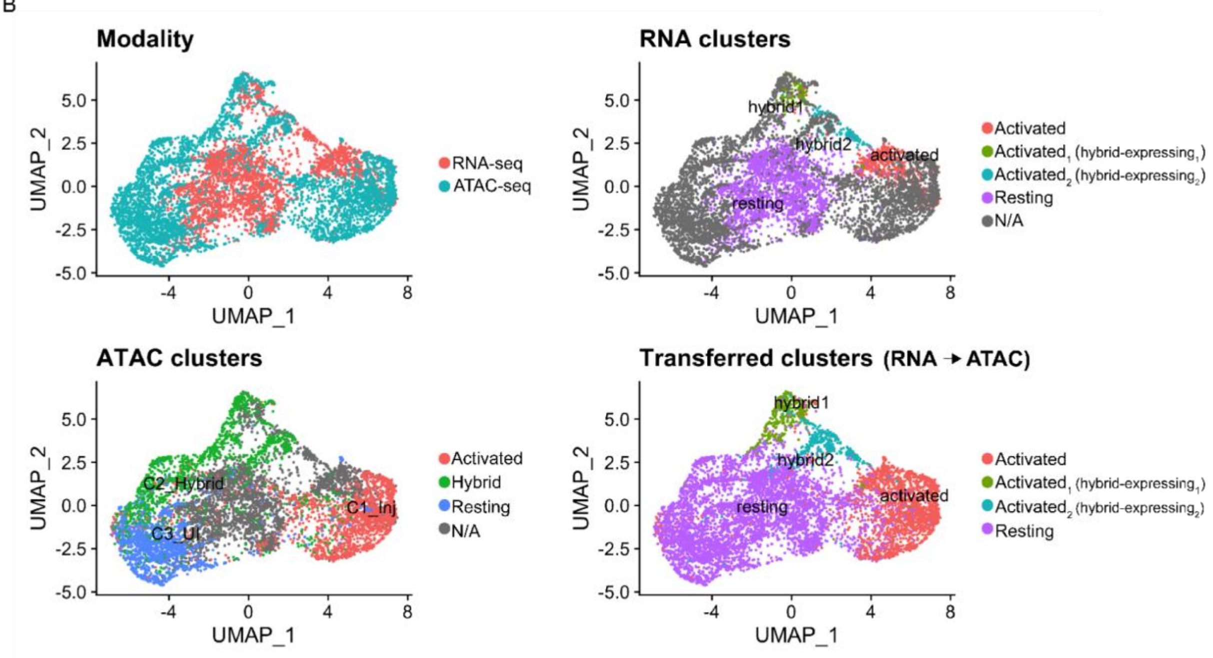

Supplemental Figure 8: The 'Hybrid' epigenomic state represents injured and uninjured cells and is marked by genes that are expressed after injury. ***(A)*** *Scaled gene activity of hybrid markers for injured versus uninjured cells in each scATAC-seq cluster. The x-axis represents the interaction of cell state (C1_Inj: injured state, C2_Hybrid: hybrid state and C3_UI: uninjured state) with treatment (Inj: injured, UI: uninjured). The y-axis represents scaled estimated gene activity scores based on the scATAC-seq data. The activity for all markers of the hybrid cluster are plotted, showing that both the uninjured and the injured cells within the hybrid cluster contribute to the hybrid markers.* ***(B)*** *Joint dimensionality reduction and cell label transfer of RNA-seq to ATAC-seq data reveals 'Activated$_1$' and 'Activated$_2$' RNA-seq subclusters, which express hybrid markers, mapped to the 'Hybrid' cluster in the ATAC-seq dataset.*

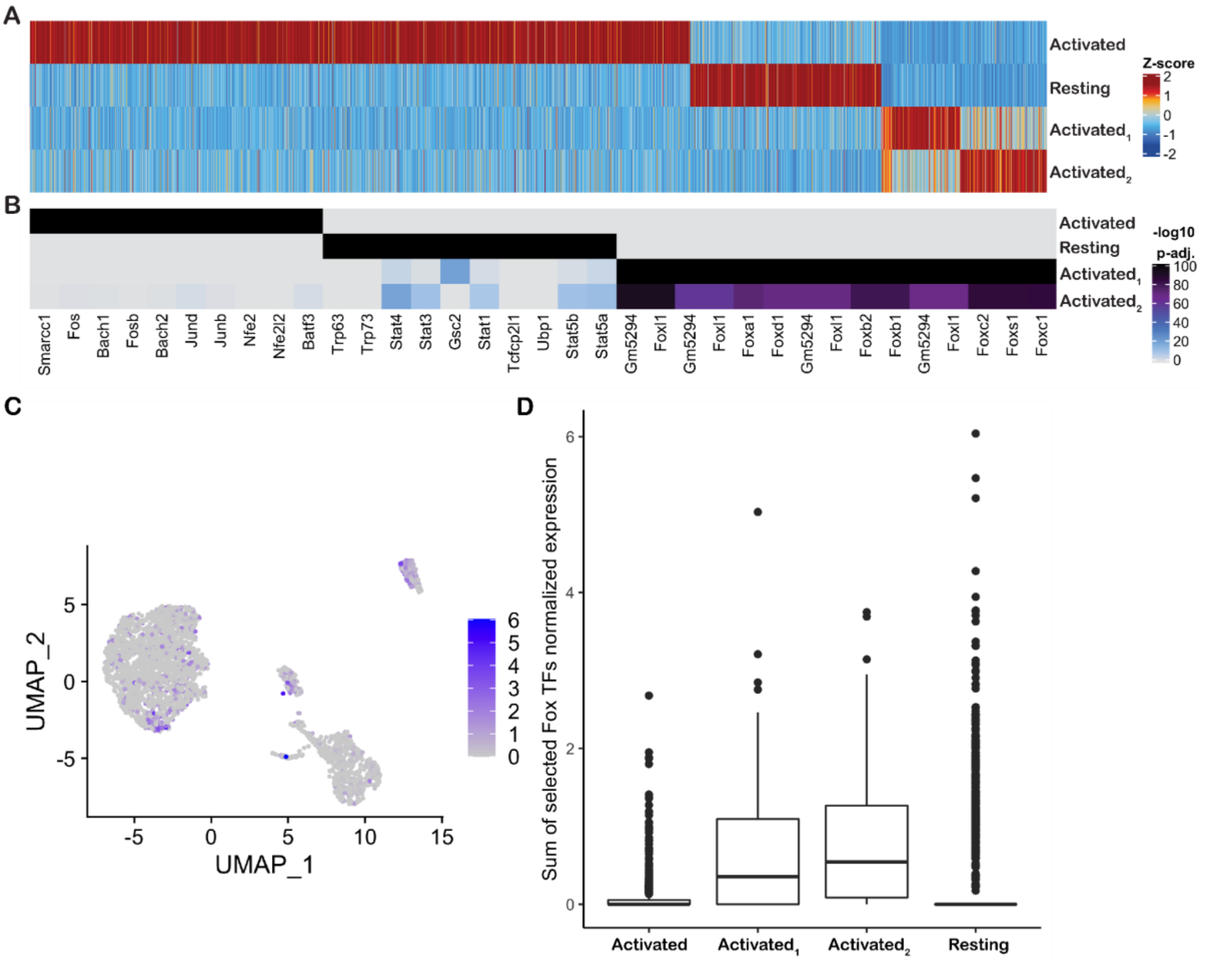

Supplemental Figure 9: Enrichment of Fox transcription factors in the Activated1 and Activated2 subclusters. ***(A)*** *Heatmap of scaled gene activity scores based on scATAC-seq data, showing markers for injured and resting clusters and two activated subclusters.* ***(B)*** *Heatmap of adjusted p-values for the top 10 most enriched transcription factor motifs in marker peaks of each of the identified scATAC-seq subclusters.* ***(C)*** *Normalized expression of Fox TFs in UMAP space. Darker color corresponds with higher gene expression.* ***(D)*** *Boxplots of the sum of normalized expression of Fox TFs across the four subclusters.*

Gene	Uninjured Median	Uninjured Mean	Injured Median	Injured Mean
Cd36	0	0.18	0	0.18
Aldh1a7	0	0.08	0	0.08
Trim46	0	0.49	0.32	0.52
Erich3	0	0.09	0	0.12
Cap2	0	0.31	0.30	0.37
Adh7	0	0.30	0	0.29

Supplemental Figure 10: Median and mean values of hybrid marker gene activity scores for uninjured versus injured cells in the hybrid cluster.
